# Decoupled dynamics of absolute and relative lymphocyte counts and age−polarized CD4^+^/CD8^+^ ratio in infants versus older adults

**DOI:** 10.3389/fimmu.2025.1599515

**Published:** 2025-07-24

**Authors:** Ting Ge, Guixin He, Qian Cui, Shuangcui Wang, Zekun Wang, Yingying Xie, Yuanyuan Tian, Juyue Zhou, Wentao Li, Baohui Wang, Keming Zhang, Jianchun Yu

**Affiliations:** ^1^ Central Laboratory, First Teaching Hospital of Tianjin University of Traditional Chinese Medicine, Tianjin, China; ^2^ Graduate School, Tianjin University of Traditional Chinese Medicine, Tianjin, China; ^3^ National Clinical Research Center for Chinese Medicine Acupuncture and Moxibustion, First Teaching Hospital of Tianjin University of Traditional Chinese Medicine, Tianjin, China; ^4^ Department of Biostatistics, School of Global Public Health, New York University, New York, NY, United States; ^5^ Department of Oncology, First Teaching Hospital of Tianjin University of Traditional Chinese Medicine, Tianjin, China; ^6^ The First Affiliated Hospital of Zhejiang Chinese Medical University, Zhejiang Chinese Medical University, Hangzhou, China; ^7^ Department of Nutrition, First Teaching Hospital of Tianjin University of Traditional Chinese Medicine, Tianjin, China

**Keywords:** peripheral blood lymphocyte subsets, absolute counts, CD4 +/CD8 + ratio, infants, older adults

## Abstract

**Background:**

Significant phenotypic and functional differences in peripheral lymphocyte subsets between infants and the elderly contribute to age-related variations in disease susceptibility and clinical outcomes. However, we are unable to specifically analyze the underlying causes owing to a lack of data on lymphocyte absolute counts and functional markers from two extremes of age.

**Methods:**

A total of 111 infants (≤ 6 months) and 111 older adults (≥ 65 years) were enrolled to assess the percentages and absolute counts of peripheral blood lymphocyte (PBL) subsets. These included CD3^+^ T cells, CD4^+^ T cells, CD8^+^ T cells, B cells, NK cells, naïve T cells (Tn), stem cell memory T cells (Tscm), central memory T cells (Tcm), effector memory T cells (Tem), and terminally differentiated effector memory T cells (Temra). Differences in PBL phenotypes by age group and gender were analyzed using the Wilcoxon rank-sum test. In addition, linear regression analysis was performed to examine the associations between the CD4^+^/CD8^+^ ratio and various PBL subsets.

**Results:**

Comparative analysis demonstrated that infants had significantly higher absolute counts of CD3^+^ T cells, CD4^+^ T cells, CD8^+^ T cells, B cells, and both CD4^+^ and CD8^+^ subsets of Tn, Tscm, Tcm, and Temra (*p* < 0.001), despite significantly lower percentages of these cell types (*p* < 0.001). In contrast, older adults exhibited reduced absolute counts but relevated percentages for all the aforementioned lymphocyte subsets, except for CD4^+^ and CD8^+^ Tn cells, which showed lower percentages (*p* < 0.001). Notably, NK cells were significantly increased in both percentage and absolute count among older adults (*p* < 0.001). The CD4^+^/CD8^+^ ratio showed marked age-related polarization, with significantly higher values in infants compared to older adults (median, 2.60 [IQR, 2.02–3.36] *vs*. 1.60 [IQR, 1.15–2.14]), a difference particularly pronounced in female infants (*p* < 0.001). Gender-related differences were observed only in the infant group, where female infants exhibited significantly higher absolute counts of CD3^+^ T cells, CD3^+^CD4^+^ T cells, and CD4^+^ Tscm and Tcm subsets (*p* < 0.05). Furthermore, linear regression analysis revealed that in infants, the CD4^+^/CD8^+^ ratio was positively associated with the percentages and absolute counts of CD4^+^ Tn cells and the percentage of CD4^+^ Tcm cells (*p* < 0.05), while showing a negative correlation with the percentages of CD8^+^ Tn and memory T (Tm) cell subsets (*p* < 0.05).

**Conclusion:**

PBL profiles exhibit marked heterogeneity at the extremes of age, with infants showing abundant naïve and memory T cell reserves, while older adults are characterized by increased NK cell activity. The age-dependent polarization of the CD4^+^/CD8^+^ ratio may serve as a potential biomarker of immunosenescence, offering valuable reference points for age-tailored vaccination strategies and immune risk stratification in the elderly.

## 1 Introduction

During the COVID-19 pandemic, notable differences in clinical responses to SARS-CoV-2 infection between infants and older adults underscored the functional heterogeneity of the immune system across the lifespan. A recent study published in *Cell (* 1) reported that infants are capable of mounting sustained antibody responses lasting up to 300 days post-infection, despite their immunological immaturity. As a result, infants exhibited a lower incidence of severe COVID-19. In contrast, older adults demonstrated increased susceptibility to severe illness, largely attributable to T cell dysfunction, chronic inflammation, and impaired memory immune responses associated with immunosenescence (2). These findings highlight fundamental differences in immune system composition and function at the extremes of age. Therefore, a comprehensive analysis of the dynamic profiles of PBL subsets during infancy and old age is essential for elucidating the mechanisms underlying immune development and aging, and for informing age-specific immunotherapeutic strategies.

The composition and function of PBL subpopulations differ markedly between infants and older adults. Shaped by the intrauterine environment, the infant immune system exhibits a heightened state of tolerance, with PBLs predominantly composed of cells bearing naïve phenotypes. In contrast, older adults frequently experience lymphopenia, characterized by reduced numbers of B and T cells (3), as well as diminished receptor gene diversity, contributing to age-related heterogeneity in both the percentages and absolute counts of PBL subsets. Absolute counts reflect the actual number of immune cells, providing insight into immune reserve capacity, whereas percentages represent the relative distribution of each subset within the total lymphocyte pool (4). The CD4^+^/CD8^+^ ratio is also a key marker of immune homeostasis, with abnormal shifts commonly associated with immunodeficiency and chronic inflammation (5). However, previous studies have primarily focused on age-associated changes in the relative proportions of PBL subsets, often overlooking the clinical relevance and dynamic nature of their absolute counts (6). This lack of absolute count data limits clinical interpretation, as a high percentage may mask critically low cell numbers, potentially leading to overestimation of immune competence (7). Moreover, many existing studies have either concentrated on a single age group (8) or adopted broad comparisons across all age ranges (9), thus failing to provide direct comparisons between infants and older adults. This gap hinders accurate assessments of immune function at the extremes of age.

In this study, high-dimensional flow cytometry was employed for the first time to comprehensively categorize PBL subsets, enabling simultaneous analysis of age- and gender-specific differences in their percentages, absolute counts, and CD4^+^/CD8^+^ ratios. Through multi-dimensional integration, we elucidated how immune reserve capacity and the balance among lymphocyte subsets collectively define age-specific immune profiles. These findings offer a novel framework for precise immune monitoring and surveillance across different life stages.

## 2 Materials and methods

### 2.1 Ethical approval

Ethical approval for the collection and analysis of blood samples from infants and older adults was obtained from the Ethics Committee of the First Teaching Hospital of Tianjin University of Traditional Chinese Medicine (Approval Nos. TYLL2021[K]001 and TYLL2023[K]033). Written informed consent was obtained from all participants in accordance with the Declaration of Helsinki, including from older adults and from the parents or legal guardians of all participating infants.

### 2.2 Study participants

This study recruited 111 infants (≤ 6 months old) (10) and 111 older adults (≥ 65 years old) (11), in Tianjin, China, between July 1 and December 31, 2024. Peripheral blood samples were collected, and immunophenotypic profiles of circulating PBL subsets were analyzed to assess age-related differences. Gender distribution was as follows: infants (male, 55; female, 56) and older adults (male, 51; female, 60). All participants were in good health, with normal blood chemistry, liver and renal function, blood glucose, and lipid profiles. None had a history of infectious diseases, hematological disorders, malignancy, or immune-related conditions. Additionally, all participants had no recent history (within four weeks) of blood transfusion, exposure to infectious individuals, or significant medication use.

### 2.3 Flow cytometry instruments and antibodies

Flow cytometry was conducted using a DxFLEX Flow Cytometer (Beckman Coulter, Jiangsu, China), and data analysis was performed with CytExpert software (version 2.4). PBL subsets—including CD3^+^ T cells, CD4^+^ T cells, CD8^+^ T cells, B cells, and NK cells (collectively known as T cells and B cells (collectively known as TBNK lymphocyte subsets)—were identified based on surface marker expression. Within CD4^+^ and CD8^+^ T cell populations, naïve T cells (Tn) and memory T cells (Tm) were further stratified (12). The Tm compartment included stem cell memory T cells (Tscm), central memory T cells (Tcm), effector memory T cells (Tem), and terminally differentiated effector memory T cells (Temra). The monoclonal antibodies used for immunophenotyping were as follows: FITC anti-human CD3 (BioLegend; clone OKT3; catalog number 317306), APC anti-human CD19 (BioLegend; clone HIB19; catalog number 302212), PE/Cyanine7 anti-human CD16 (BioLegend; clone 3G8; catalog number 302016), PE/Cyanine7 anti-human CD56 (NCAM) (BioLegend; clone HCD56; catalog number 318318), PerCP/Cyanine5.5 anti-human CD4 (BioLegend; clone RPA-T4; catalog number 300530), Brilliant Violet 510™ anti-human CD8 (BioLegend; clone SK1; catalog number 344732), APC/Fire™ 750 anti-human CD45 (BioLegend; clone HI30; catalog number 304062), PE anti-human CD45RA (BioLegend; clone HI100; catalog number 304108), Brilliant Violet 421™ anti-human CD197 (CCR7) (BioLegend; clone G043H7; catalog number 353208), and Brilliant Violet 605™ anti-human CD95 (Fas) (BioLegend; clone DX2; catalog number 305628).

### 2.4 Lymphocyte subsets enumeration and phenotyping

**Figure 1:**
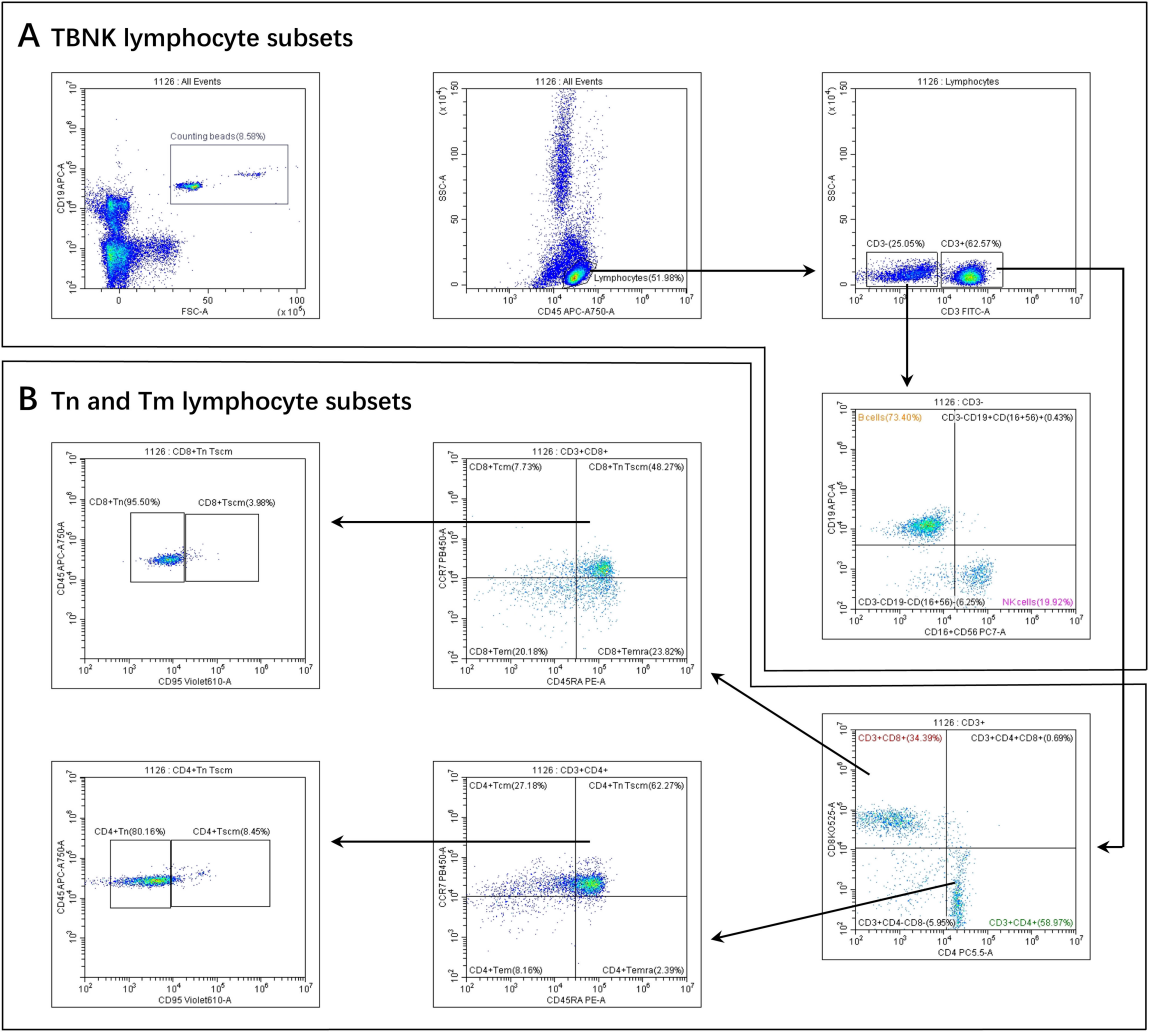
Gating strategies for PBL subsets. **(A)** TBNK lymphocyte subsets. **(B)** Tn and Tm lymphocyte subsets. Firstly, we gated Beads. At the same time, we gated lymphocytes identified by CD45 from a leukocyte, then gated CD3^+^ T cells and CD3^-^ T cells from a lymphocyte.Secondly, From the CD3^-^ T cells, B cells and NK cells were selected. From the CD3^+^ T cells, CD4^+^ T cells and CD8^+^ T cells were selected. Thirdly, CD4^+^ Tcm, CD4^+^ Tem, CD4^+^ Temra, and CD4^+^ TnTscm lymphocyte subsets were gated from the CD4^+^ T cells. Next, CD4^+^ Tn and CD4^+^ Tscm were gated from the CD4^+^ TnTscm lymphocyte subsets. The gating logic for each subset of CD8^+^ T cells was the same as above.

A total of 500 μL of peripheral blood was collected from healthy infant and older adult volunteers using EDTA anticoagulation tubes. Samples were gently mixed and promptly transported to the laboratory for flow cytometric analysis. The operational procedure was as follows. Flow cytometry tubes were prepared according to the number of samples, each labeled with a uniform identifier. To each tube, 5 μL of a monoclonal antibody cocktail—comprising anti-CD3, CD19, CD16, CD56, CD4, CD8, CD45, CD45RA, CD197 (CCR7), and CD95—was added. Subsequently, 50 μL of well-mixed, anticoagulated whole blood was added to each tube. After incubation for 15 minutes at room temperature, protected from light, 450 μL of red blood cell lysis buffer was added. Tubes were mixed and incubated again for 15 minutes at room temperature. Finally, 50 μL of vortexed absolute counting beads were added. The samples were then analyzed by flow cytometry to determine the percentages of lymphocyte subpopulations, and absolute cell counts were calculated using the formula: cells/μL = (acquired cells × total beads)/(acquired beads × sample volume). Cell populations were defined as follows: T cells were identified as CD3^+^ cells; B cells as CD3^-^CD19^+^ cells; NK cells as CD3^+^/^-^CD16^+^CD56^+^ cells; CD4^+^ T cells as CD3^+^CD4^+^ cells; and CD8^+^ T cells as CD3^+^CD8^+^ cells. Tn cells were defined as CD45RA^+^CCR7^+^CD45^+^CD95^-^; Tscm cells as CD45RA^+^CCR7^+^CD45^+^CD95^+^; Tcm cells as CD45RA^-^CCR7^+^; Tem cells as CD45RA^-^CCR7^-^; and Temra cells as CD45RA^+^CCR7^-^. The complete flow cytometry gating strategy used for the identification of these subsets is presented in **Figure 1**.

### 2.5 Statistical analysis

All statistical analyses were performed using R software (version 4.2.1). Heatmaps of PBL subset distributions were generated using the ComplexHeatmap R package (13). Descriptive statistics were reported for both continuous and categorical variables. For continuous variables, the median and interquartile range (IQR) were calculated; for categorical variables, frequencies were provided. The Shapiro–Wilk test was used to assess normality, and Levene’s test was applied to evaluate homogeneity of variance. Group comparisons for continuous variables were conducted using the Wilcoxon rank-sum test. Linear regression analysis was employed to assess associations between the CD4^+^/CD8^+^ ratio and lymphocyte subset counts. A *p*-value < 0.05 was considered statistically significant. In all figures, statistical significance was indicated as follows: ***p < 0.001; **p < 0.01; *p < 0.05.

## 3 Results

### 3.1 General characteristics

**Figure 2:**
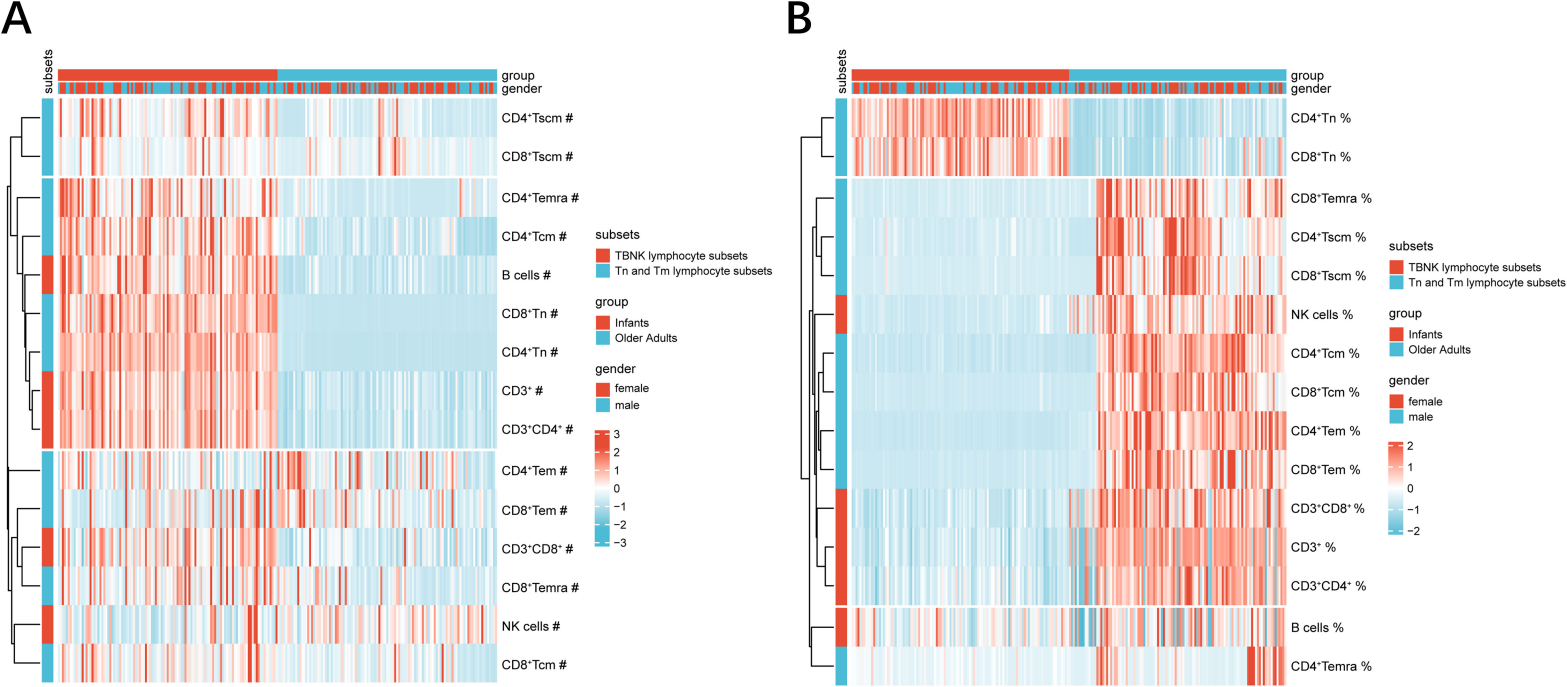
PBL subsets heatmaps at extremes of age. **(A)** The heatmap represented the absolute counts of PBL subsets. **(B)** The heatmap represented the percentages of PBL subsets. The color of the heatmaps indicated percent heteroplasmy, where blue and red represent low–and high–subset frequency, respectively. Hierarchical clustering: vertical axis, PBL subsets; horizontal axis, group and gender. absolute counts and percentages were signified by ‘#’ symbols and ‘%’ symbols, respectively.

Descriptive summary statistics for infants and older adults, stratified by sex, are presented in **Table 1**. The proportion of males and females was approximately balanced within each age group. In addition to reporting the median percentages and absolute counts of T cells, B cells, NK cells, and CD4^+^/CD8^+^ Tn and Tm subsets, the table also includes the median CD4^+^/CD8^+^ ratio for each group. Heatmap analysis revealed that infants exhibited higher absolute counts of CD3^+^ T cells, CD4^+^ and CD8^+^ T cell subsets (including Tn, Tscm, Tcm, and Temra), and B cells compared to older adults, while NK cells showed an opposite trend (**Figure 2A**). In contrast, the percentages of CD3^+^, CD4^+^, CD8^+^, B, and NK cells—as well as CD4^+^ and CD8^+^ Tscm, Tcm, and Temra subsets—were lower in infants, with the exception of Tn cells, which were elevated in both CD4^+^ and CD8^+^ subsets (**Figure 2B**). However, further statistical testing was required to determine whether these differences were significant between the two age groups.

**Table 1:** The distribution of peripheral lymphocyte subsets in the infants and the older adults.

Parameters	All	Infants (≤6 months)	Older Adults (≥65 years)	*P* value
	n=222	n=111	n=111	
gender	116:106	56:55	60:51	0.687
CD3^+^ (cells/µl)	2158.73 (1623.71 – 3849.86)	3852.76 (3128.37 – 4678.74)	1628.00 (1159.50 – 2033.00)	< 0.001
CD3^+^CD4^+^ (cells/µl)	1563.56 (883.90 – 2420.61)	2426.16 (2072.40 – 3044.33)	891.00 (628.00 – 1139.00)	< 0.001
CD3^+^CD8^+^ (cells/µl)	748.00 (534.45 – 999.25)	971.20 (724.51 – 1378.21)	589.00 (450.00 – 754.83)	< 0.001
B cells (cells/µl)	530.64 (251.50 – 1041.83)	1043.64 (839.46 – 1429.91)	259.00 (162.31 – 364.00)	< 0.001
NK cells (cells/µl)	303.12 (188.00 – 420.00)	229.77 (159.29 – 360.16)	365.00 (265.00 – 445.17)	< 0.001
CD4^+^Tn (cells/µl)	455.23 (18.08 – 1415.75)	1416.93 (1141.96 – 1783.45)	18.03 (9.60 – 33.16)	< 0.001
CD4^+^Tscm (cells/µl)	110.70 (39.98 – 203.82)	189.63 (121.38 – 280.29)	48.23 (20.99 – 96.50)	< 0.001
CD4+Tcm (cells/µl)	289.56 (157.24 – 444.84)	443.95 (338.73 – 627.69)	163.25 (109.17 – 220.55)	< 0.001
CD4^+^Tem (cells/µl)	136.97 (96.58 – 195.41)	142.79 (98.99 – 188.45)	125.96 (88.99 – 197.67)	0.703
CD4^+^Temra (cells/µl)	23.06 (3.72 – 56.74)	52.44 (28.72 – 99.47)	4.51 (1.10 – 15.07)	< 0.001
CD8^+^Tn (cells/µl)	59.09 (4.97 – 604.14)	604.17 (444.58 – 751.51)	4.95 (2.27 – 11.86)	< 0.001
CD8^+^Tscm (cells/µl)	25.76 (12.50 – 48.31)	30.71 (18.52 – 56.51)	18.26 (8.10 – 37.64)	< 0.001
CD8^+^Tcm (cells/µl)	49.93 (33.74 – 82.43)	72.35 (41.68 – 98.89)	36.65 (23.10 – 61.20)	< 0.001
CD8^+^Tem (cells/µl)	78.53 (41.14 – 175.00)	66.96 (35.94 – 183.53)	85.74 (46.70 – 163.16)	0.352
CD8^+^Temra (cells/µl)	73.59 (41.18 – 156.87)	111.64 (64.09 – 233.52)	53.23 (25.23 – 104.49)	< 0.001
CD3^+^ % lymphocyte cells	40.43% (32.16 – 69.75%)	35.17% (29.79 – 38.88%)	70.00% (60.00 – 74.00%)	< 0.001
CD3^+^CD4^+^ % CD3+	27.00% (20.54 – 38.00%)	22.07% (19.46 – 25.39%)	38.00% (31.00 – 44.83%)	< 0.001
CD3^+^CD8^+^ % CD3+	12.42% (8.19 – 24.00%)	8.72% (7.00 – 11.12%)	24.00% (19.00 – 31.00%)	< 0.001
B cells % lymphocyte cells	10.85% (8.00 - 12.58%)	9.79% (8.11 – 11.58%)	11.00% (8.00 – 14.00%)	0.016
NK cells % lymphocyte cells	4.23% (1.99 – 14.75%)	2.08% (1.49 – 3.03%)	15.00% (9.17 – 20.00%)	< 0.001
CD4^+^Tn % CD3^+^CD4^+^	7.65% (2.73 - 12.59%)	12.62% (10.34 – 15.55%)	2.70% (1.52– 4.48%)	< 0.001
CD4^+^Tscm % CD3^+^CD4^+^	3.01% (1.21 – 9.48%)	1.57% (1.07 – 2.53%)	9.40% (3.75 – 17.25%)	< 0.001
CD4^+^Tcm % CD3^+^CD4^+^	5.99% (3.87 – 25.28%)	4.11% (3.43 – 4.95%)	25.30% (19.93 – 33.25%)	< 0.001
CD4^+^Tem % CD3^+^CD4^+^	2.42% (1.32 – 20.00%)	1.32% (0.80– 1.79%)	20.00% (14.73 – 29.53%)	< 0.001
CD4^+^Temra % CD3^+^CD4^+^	0.46% (0.20 - 1.05%)	0.46% (0.27 – 0.85%)	0.40% (0.10 – 2.28%)	0.913
CD8^+^Tn % CD3^+^CD8^+^	3.34% (0.80 - 5.38%)	5.21% (3.90 – 6.75%)	0.80% (0.41 – 2.20%)	< 0.001
CD8^+^Tscm % CD3^+^CD8^+^	0.70% (0.21 - 5.70%)	0.27% (0.16 – 0.52%)	5.40% (1.62 – 11.55%)	< 0.001
CD8^+^Tcm % CD3^+^CD8^+^	1.12% (0.52 - 10.35%)	0.63% (0.40 – 0.85%)	10.50% (5.20 – 14.13%)	< 0.001
CD8^+^Tem % CD3^+^CD8^+^	2.61% (0.57 – 22.05%)	0.58% (0.36 – 1.62%)	22.30% (8.67 – 34.68%)	< 0.001
CD8^+^Temra % CD3^+^CD8^+^	2.45% (0.88 – 10.20%)	0.91% (0.60 – 2.12%)	10.20% (4.85 – 22.47%)	< 0.001
CD4^+^/CD8^+^	2.06 (1.45 - 2.76)	2.60 (2.02 – 3.36)	1.60 (1.15 – 2.14)	< 0.001

Data are presented as female: male or median (IQR). p < 0.05 was considered significant.

### 3.2 Absolute counts and percentages of TBNK lymphocyte subsets at age extremes

**Figure 3:**
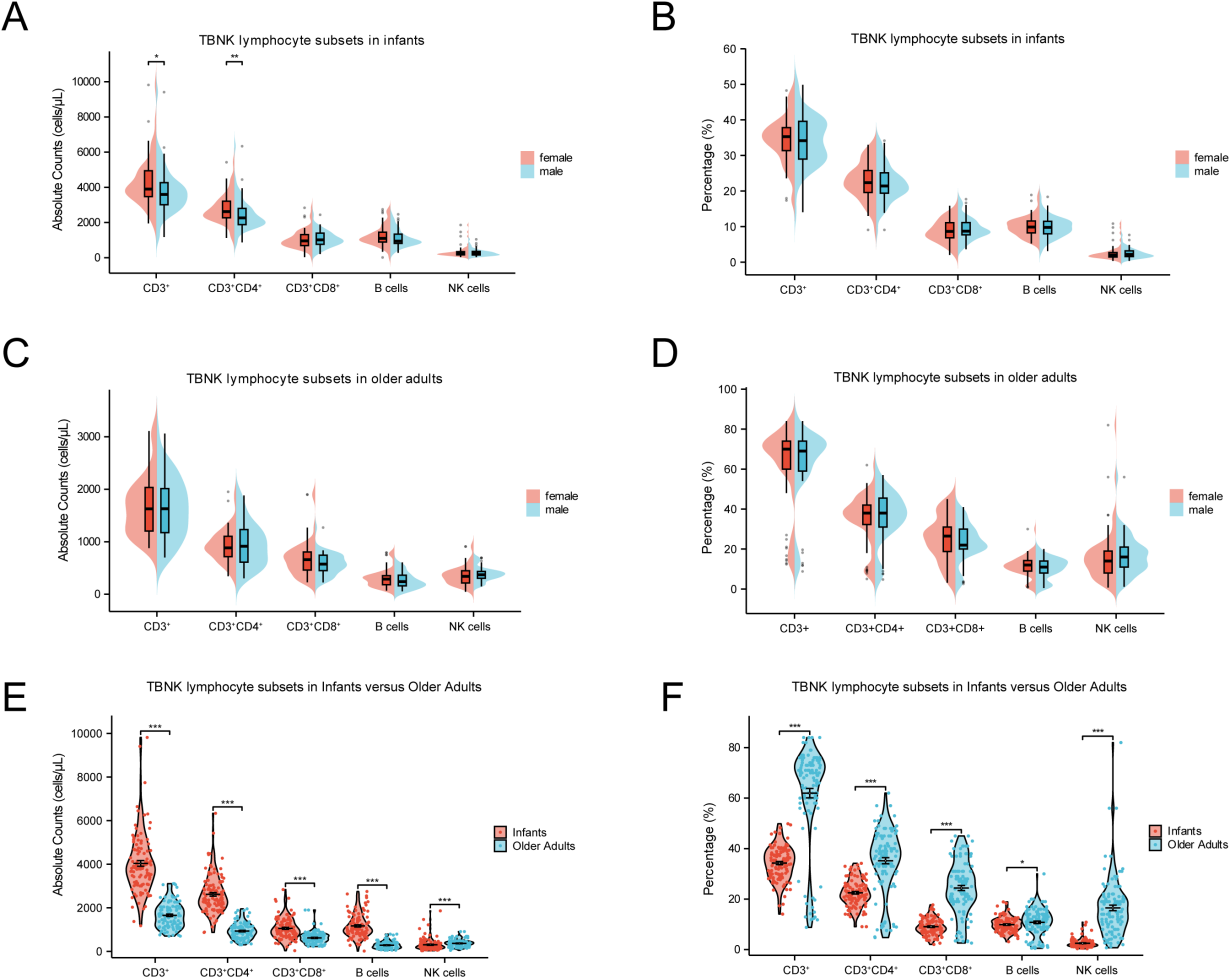
Comparisons of TBNK lymphocyte subsets at extremes of age. **(A)** Comparison of the absolute counts between female and male in infants. **(B)** Comparison of percentages between female and male in infants. **(C)** Comparison of the absolute counts between female and male in older adults. **(D)** Comparison of percentages between female and male in older adults. **(E)** Comparison of the absolute counts between infants and older adults. **(F)** Comparison of percentages between infants and older adults. (***p < 0.001; **p < 0.01; *p < 0.05).

In this study, we first examined the influence of gender on the percentages and absolute counts of TBNK lymphocyte subsets at the extremes of age. As shown in **Figures 3A, B**, female infants exhibited significantly higher absolute counts of CD3^+^ T cells (*p* < 0.05) and CD4^+^ T cells (*p* < 0.01) compared to male infants, while no significant differences were observed in the corresponding percentages. A similar trend was observed among older adults; however, neither the percentages nor the absolute counts of TBNK subsets showed statistically significant gender differences in this group (**Figures 3C, D**). These findings suggest that, unlike in infancy, the distribution of TBNK lymphocyte subsets remains largely balanced between older males and females.

Age emerged as a critical determinant influencing both the percentages and absolute counts of TBNK lymphocyte subsets. As shown in **Figures 3E, F**, infants exhibited significantly higher absolute counts of CD3^+^ T cells, CD4^+^ T cells, CD8^+^ T cells, and B cells compared to older adults (*p* < 0.001). In contrast, the percentages of these same subsets were markedly lower in infants (*p* < 0.001). Regarding innate immune cells, both the percentage and absolute count of NK cells were significantly lower in infants than in older adults (*p* < 0.001). These results underscore substantial age-related compositional shifts in TBNK lymphocyte subsets, which may reflect underlying biological mechanisms associated with immune development in early life and immunosenescence in later life.

### 3.3 Absolute counts and percentages of Tn and Tm lymphocyte subsets at age extremes

**Figure 4:**
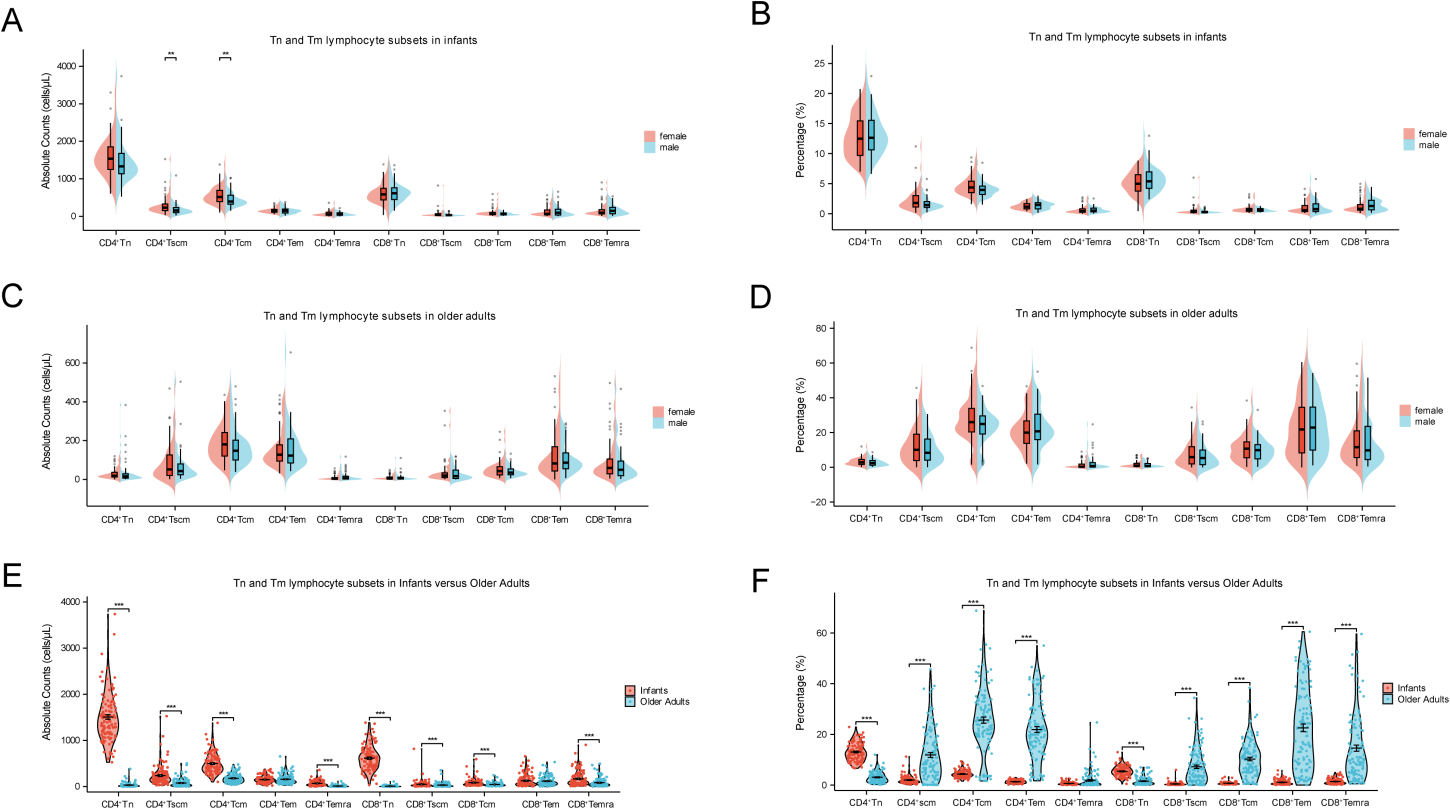
Comparisons of Tn and Tm lymphocyte subsets at extremes of age. **(A)** Comparison of the absolute counts between female and male in infants. **(B)** Comparison of percentages between female and male in infants. **(C)** Comparison of the absolute counts between female and male in older adults. **(D)** Comparison of percentages between female and male in older adults. **(E)** Comparison of the absolute counts between infants and older adults. **(F)** Comparison of percentages between infants and older adults. (***p < 0.001; **p < 0.01; *p < 0.05).

Tn and Tm lymphocyte subsets play pivotal roles in immune function, with Tn cells initiating immune responses and Tm cells mediating immunological memory. As shown in **Figures 3A–D**, gender had no significant effect on the percentages or absolute counts of most Tn and Tm lymphocyte subsets. However, female infants exhibited significantly higher absolute counts of CD4^+^ Tscm and CD4^+^ Tcm cells compared to male infants (*p* < 0.001). In contrast, age exerted a more substantial influence on Tn and Tm lymphocyte subsets distribution. As depicted in **Figures 4E, F**, both the absolute counts and percentages of CD4^+^ Tn and CD8^+^ Tn cells were markedly reduced in older adults (*p* < 0.001), consistent with age-related immune decline and immunosenescence. Conversely, the percentages of Tm subsets—including CD4^+^ Tscm, CD4^+^ Tcm, CD4^+^ Tem, CD8^+^ Tscm, CD8^+^ Tcm, CD8^+^ Tem, and CD8^+^ Temra—were significantly elevated in older adults (*p* < 0.001).

### 3.4 CD4^+^/CD8^+^ ratio at age extremes

**Figure 5:**
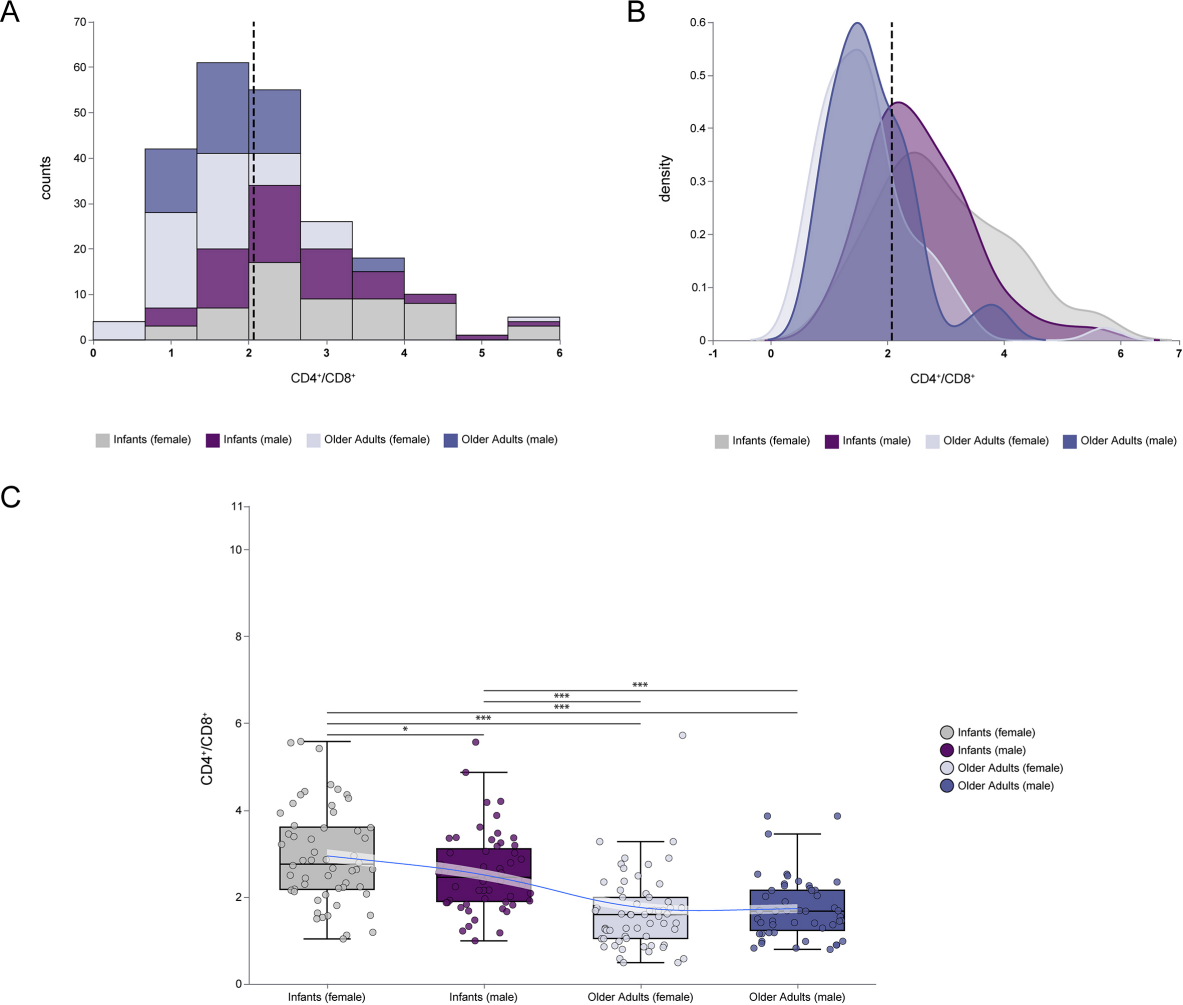
Comparisons of CD4^+^/CD8^+^ ratio at extremes of age. **(A)** The frequency histogram of CD4^+^/CD8^+^ ratio at extremes of age. **(B)** The density plot of CD4^+^/CD8^+^ ratio at extremes of age. **(C)** Comparison of CD4^+^/CD8^+^ ratio between infants and older adults, including female and male in each group. The frequency histograms described the data distribution of individual variables. The density plot described the concentrating and dispersing trends of the data. The vertical dashed line represented the median value of all data. (***p < 0.001; **p < 0.01; *p < 0.05).

The CD4^+^/CD8^+^ ratio is a key indicator of immune homeostasis and can reflect distinct immunological states at the extremes of age. As shown in **Figure 5A**, infants exhibited a generally higher CD4^+^/CD8^+^ ratio compared to older adults, with female infants displaying slightly higher values than male infants. **Figure 5B** further illustrates a right-shifted distribution curve in the infant group (regardless of gender), indicating a significantly elevated CD4^+^/CD8^+^ ratio relative to older adults. In **Figure 5C**, detailed comparison of age and gender effects revealed that the CD4^+^/CD8^+^ ratio was significantly higher in infants than in older adults (median: 2.60 [IQR: 2.02–3.36] *vs*. 1.60 [IQR: 1.15–2.14], *p* < 0.001), with the most pronounced elevation observed in female infants. These findings suggest that age is the primary determinant of CD4^+^/CD8^+^ ratio variation, while gender influences this parameter significantly in infants (*p* < 0.05), but not in older adults.

**Figure 6:**
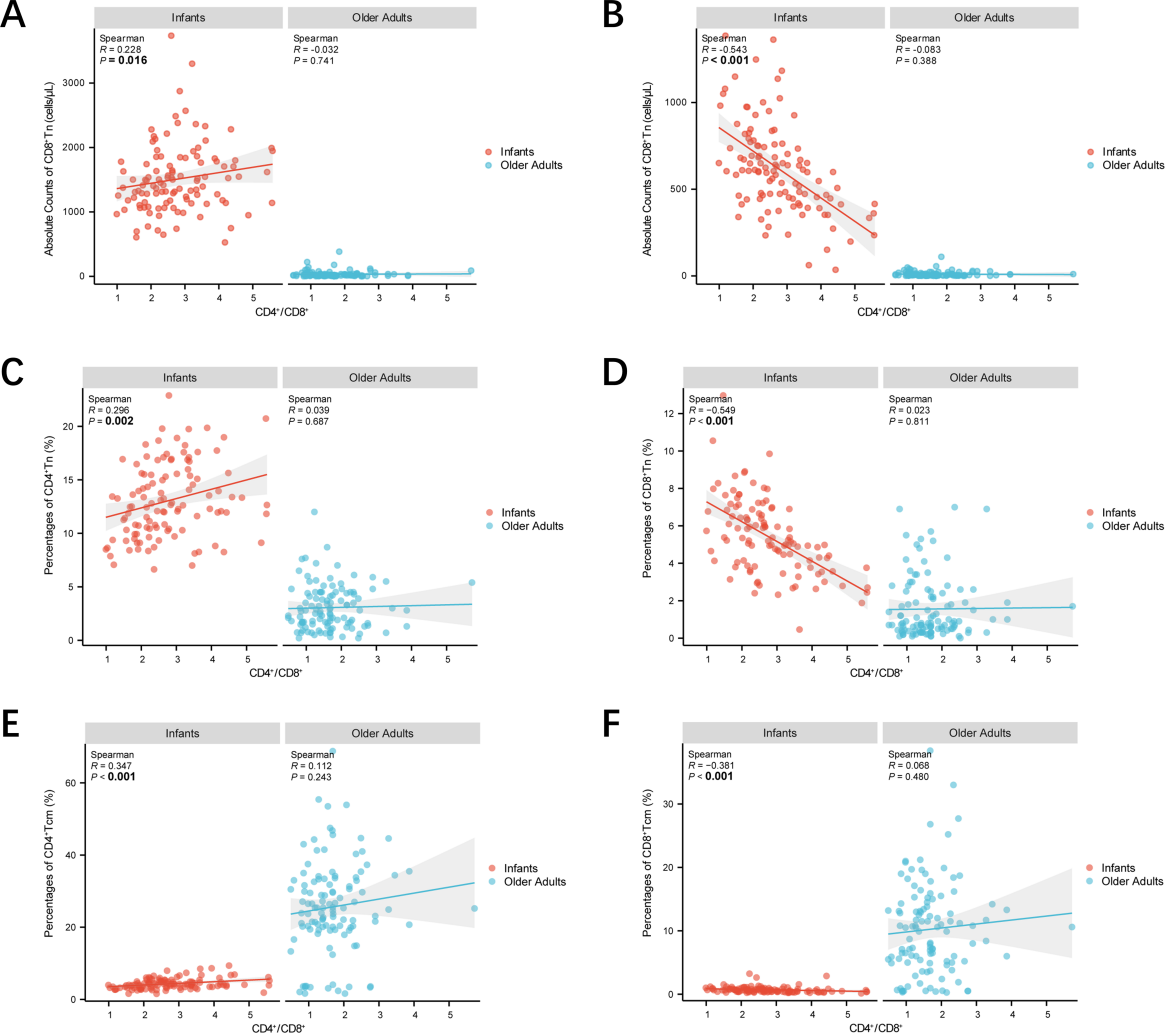
Scatter plots demonstrating the correlations between CD4^+^/CD8^+^ ratio and Tn and Tm lymphocyte subsets at extremes of age. **(A)** Scatter plots demonstrating CD4^+^/CD8^+^ ratio—absolute counts of CD4^+^ Tn correlations. **(B)** Scatter plots demonstrating CD4^+^/CD8^+^ ratio—absolute counts of CD8^+^ Tn correlations. **(C)** Scatter plots demonstrating CD4^+^/CD8^+^ ratio—percentages of CD4^+^ Tn correlations. **(D)** Scatter plots demonstrating CD4^+^/CD8^+^ ratio—percentages of CD8^+^ Tn correlations. **(A)** Scatter plots demonstrating CD4^+^/CD8^+^ ratio—percentages of CD4^+^ Tcm correlations. **(B)** Scatter plots demonstrating CD4^+^/CD8^+^ ratio—percentages of CD8^+^ Tcm correlations. Correlations were assessed by scatter plot, and the strength of linear correlation was determined by calculating a Pearsonman coefficient. Infants were represented by red dots and older adults by blue dots. The shaded line around each linear fit line represented 95% confidence interval. (***p < 0.001; **p < 0.01; *p < 0.05).

To explore the association between Tn and Tm lymphocyte subsets and the CD4^+^/CD8^+^ ratio at the extremes of age, linear regression analyses were conducted with appropriate model specifications. As shown in **Figure 6**, scatter plots and linear regression analyses were used to assess relationships between CD4^+^ Tn and Tm lymphocyte subsets and the CD4^+^/CD8^+^ ratio in both age groups. In infants, the absolute counts and percentages of CD4^+^ Tn cells, as well as the percentages of CD4^+^ Tcm cells, were significantly positively correlated with the CD4^+^/CD8^+^ ratio (*p* < 0.05). Conversely, in the same group, both the absolute counts and percentages of CD8^+^ Tn cells and the percentages of CD8^+^ Tcm cells were significantly negatively correlated with the CD4^+^/CD8^+^ ratio (*p* < 0.05). No significant correlations were observed in older adults. Additionally, **Supplementary Figure 1** shows that the absolute counts and percentages of CD8^+^ Tscm, CD8^+^ Tem, and CD8^+^ Temra cells in infants were also significantly negatively correlated with the CD4^+^/CD8^+^ ratio (*p* < 0.05). These results highlight the complexity of immune regulatory dynamics during infancy.

## 4 Discussion

This study investigated the characteristics of PBL subsets in infants and older adults, revealing significant differences in percentages, absolute counts, and the CD4^+^/CD8^+^ ratio. Peripheral blood, as the most accessible and well-characterized tissue in systems immunology, has long served as a critical window into immune function across the human lifespan (14, 15). Recent advances in technologies such as high-dimensional flow cytometry have enabled deeper profiling of immune system dynamics across age groups (16). However, the distinct immune phenotypes at the extremes of age remain incompletely understood. The first six months of life represent a critical window of immune maturation, during which PBL subsets are highly abundant and functionally active. Notably, maternal mRNA COVID-19 vaccination has been shown to enhance infant immunity through transplacental antibody transfer (17). In contrast, older adults exhibit signs of immune senescence, including reduced lymphocyte counts and impaired function, largely due to thymic involution and chronic antigenic stimulation (18). Our findings are consistent with previous work by Erkeller-Yuksel et al. (19) on age-related lymphocyte decline but extend these observations by incorporating detailed phenotyping of Tn, Tscm, Tcm, Tem, and Temra subsets, as well as analyzing sex-specific differences in infancy and the age-associated dynamics of the CD4^+^/CD8^+^ ratio as a marker of immunosenescence. To our knowledge, this study provides the first comprehensive analysis of PBL subset phenotypes at the extremes of age using high-dimensional flow cytometry.

### 4.1 Age−related differences in PBL subsets

In this study, we identified a decoupling between the percentages and absolute counts of PBL subsets in individuals at the extremes of age, a phenomenon that may obscure the true immune status. Specifically, infants exhibited significantly higher absolute counts of TBNK lymphocyte subsets compared to older adults, despite having generally lower percentages. This pattern aligns with the “clonal expansion” characteristic of early immune development in infancy (20) and the so-called “twilight of immunity” observed in the elderly (21). The clonal expansion of lymphocytes in infants provides the cellular basis for immune maturation, enabling the developing immune system to adapt to novel environmental exposures and respond effectively to a wide range of pathogens. Additionally, we found that CD4^+^ and CD8^+^ Tn cells were markedly enriched in infancy, while older adults demonstrated a compensatory increase in the percentages of memory T cell subsets, including Tscm, Tcm, and Tem. These findings support the notion that the decline in absolute counts of Tn cells serves as a hallmark of immunosenescence, consistent with the observations reported by Mittelbrunn et al. (22).

Notably, both the percentages and absolute counts of NK cells were significantly higher in older adults compared to infants. This increase may be attributed to two key factors. First, chronic antigenic stimulation—such as persistent cytomegalovirus (CMV) infection—commonly observed in older adults, can drive sustained activation and expansion of NK cells. Second, as components of the innate immune system, NK cells are relatively unaffected by age-related thymic involution, resulting in their higher representation in the peripheral blood of elderly individuals (23). Although our study did not detect significant gender-based differences in NK cell counts, we did not assess potential functional differences related to gender dimorphism. Prior research has shown that elderly males may exhibit enhanced pro-inflammatory activity within NK2 subsets compared to age-matched females (24).

### 4.2 CD4^+^/CD8^+^ ratio dynamics and implications

As a key biomarker of immune homeostasis, the CD4^+^/CD8^+^ ratio holds significant clinical value and is commonly used as an auxiliary diagnostic indicator, particularly for HIV infection in infants under two years of age (25). In this study, we observed a pronounced age-dependent polarization of the CD4^+^/CD8^+^ ratio: infants exhibited a markedly elevated ratio, whereas older adults showed a substantially reduced or even inverted ratio. This trajectory reflects a coherent immunological progression across the lifespan. In infancy, the high CD4^+^/CD8^+^ ratio is maintained by robust thymic output and a predominance of CD4^+^ Tn and CD4^+^ Tcm subsets. Our data revealed positive correlations between the CD4^+^/CD8^+^ ratio and both CD4^+^ Tn and CD4^+^ Tcm percentages, suggesting that active thymopoiesis and early-stage memory differentiation contribute to CD4^+^ T cell dominance during this period. Functionally, this likely supports the developmental requirement for immune flexibility and protection against novel antigens (26). In contrast, aging is accompanied by thymic involution and diminished production of naïve T cells, particularly within the CD4^+^ compartment. Concurrently, chronic antigenic stimulation—most notably from persistent infections such as CMV—drives the expansion of highly differentiated CD8^+^ T cells, including Temra subsets (27). This shift in T cell composition contributes to the progressive decline and possible inversion of the CD4^+^/CD8^+^ ratio in older adults, a recognized hallmark of immunosenescence (28), which has been associated with heightened susceptibility to infection and increased all-cause mortality (29). Although our data did not show a significant correlation between CD8^+^ Tm subsets and the CD4^+^/CD8^+^ ratio in older adults, this may reflect inter-individual variability in lifelong antigen exposure and infection history (30).

Moreover, the CD4^+^/CD8^+^ ratio has been linked to T cell activation status. A lower ratio is often associated with heightened T cell activation—characterized, for example, by increased frequencies of CD38^+^HLA-DR^+^CD4^+^ T cells—indicating a shift toward a more activated or potentially exhausted immune state (31). Thus, beyond its role as a phenotypic marker, the CD4^+^/CD8^+^ ratio may serve as a functional indicator of immune resilience or decline throughout the lifespan. Future studies should investigate how this ratio influences T cell activation thresholds at different developmental stages to better elucidate age-specific immune responsiveness. In light of this, we plan to further examine the impact of the CD4^+^/CD8^+^ ratio on T cell activation thresholds in individuals at extreme ages, aiming to uncover the mechanisms underlying immune heterogeneity. While our current study did not observe CD4^+^/CD8^+^ ratio inversion, it establishes a reference range and highlights age-related differences in healthy individuals at opposite ends of the age spectrum. Longitudinal and functional studies will be essential to clarify the clinical significance of this polarization and its implications for immune competence across the human lifespan.

### 4.3 Sex−based immune differences

The results of this study demonstrate significant heterogeneity in PBL phenotypes among individuals at the extremes of age. However, the role of gender in shaping these immune characteristics remains less well understood. To address this, we performed a gender-stratified analysis within the infant and older adult groups. Notably, female infants exhibited significantly higher absolute counts of CD3^+^ T cells, CD4^+^ T cells, CD4^+^ Tscm, and CD4^+^ Tcm subsets compared to male infants. These differences may reflect the influence of sex hormones on thymic output (32) and the dosage effects of immune-related genes encoded on the X chromosome (33). This observation is consistent with previous studies suggesting that females undergo faster immune maturation than males (34). To further understand the mechanisms underlying these sex-based differences, we reviewed literature on the hormonal and genetic regulation of immune development. The X chromosome harbors numerous immune-related genes, including those encoding Toll-like receptors, cytokine receptors, and factors critical for T and B cell function, as well as transcriptional and translational regulators (34). Historically, Calzolari first proposed a link between sex hormones and immune function in 1898, when he observed thymic enlargement in immature rabbits following castration (35). In addition, females have been shown to possess higher numbers of CD4^+^ T cells and elevated levels of circulating immunoglobulins, particularly IgM. Interestingly, the immune profile of male infants observed in our study may indirectly support the evolutionary hypothesis that male physiology prioritizes reproductive success over immune investment (36). While androgen signaling plays a critical role in the development of reproductive organs, it has been shown to compromise systemic immune function by inhibiting thymic T cell maturation (37). Recent studies further suggest that androgens can promote CD8^+^ T cell exhaustion through regulation of the PD-1 pathway, thereby impairing anti-tumor immunity (38). Sex steroid hormones are believed to contribute to sex-based differences in both humoral and cellular immune responses to infection and vaccination (39). Although hormonal levels were not measured in our study, these findings provide a plausible mechanistic basis for the observed immune differences between male and female infants and highlight the need for future functional studies incorporating endocrine parameters. In contrast, no significant gender differences in immune parameters were observed among older adults. This may be attributed to age-related declines in sex hormone levels—such as reductions in estrogen and testosterone—and the broader effects of immunosenescence, both of which likely diminish the regulatory influence of sex on immune homeostasis in late life (40). Notably, the gender-specific elevation of the CD4^+^/CD8^+^ ratio observed in female infants further supports the notion that sex-based immune differences are most prominent during early life. Taken together, these findings suggest that the influence of gender on immune function gradually wanes with age, reinforcing the dominant role of aging in immune system remodeling (41).

### 4.4 Study limitations and future work

Although this study provides a comprehensive characterization of peripheral blood lymphocyte subsets at the extremes of age, several limitations should be acknowledged. First, the sample size was limited to healthy individuals, and future research should include larger, more diverse cohorts encompassing various health conditions to enhance the generalizability of these findings (42). Second, our analysis focused exclusively on circulating lymphocyte subsets, without evaluating tissue-resident immune cells. Investigating their distribution and relationship with peripheral counterparts would provide a more complete picture of systemic immune regulation (43). Third, the cross-sectional design precludes causal inferences regarding age-related immune trajectories. Longitudinal studies following individuals from infancy to old age are needed to delineate dynamic changes in Tn and Tm lymphocyte subsets over time. Fourth, unmeasured confounding factors—such as maternal antibody transfer in infants or CMV serostatus in older adults—may influence the observed subset distributions. CMV seropositivity in older adults is known to drive expansion of Temra cells and reduction of the Tn compartment, while maternal antibodies may transiently modulate immune activation or suppress endogenous lymphocyte maturation in early life. Fifth, although we report phenotypic differences suggestive of immune senescence, such as Tn depletion and NK cell expansion, the lack of functional assays (e.g., cytokine secretion, proliferation capacity, activation/exhaustion markers, and TCR repertoire analysis) limits mechanistic interpretation. Lastly, while a significant increase in total NK cell counts was observed in older adults, detailed phenotyping (e.g., CD56^bright^ *vs*. CD56^dim^ subsets and expression of cytotoxic markers) was not performed, which may obscure functional heterogeneity within the NK compartment. Therefore, future studies should prioritize the integration of absolute counts of PBL subsets into clinical algorithms to support applications such as optimizing age-specific vaccination schedules in infants and stratifying immune risk in older adults. Functional validation of phenotypic findings is also essential and should include assays assessing cytokine secretion, cell proliferation, activation and exhaustion markers, T cell receptor (TCR) repertoire diversity, and detailed NK cell profiling using markers such as CD107a, CD56^bright^, CD56^dim^, and IFN-γ expression. Importantly, these immunological parameters should be evaluated in relation to clinical outcomes—such as vaccine responsiveness or susceptibility to infection—to elucidate their mechanistic relevance and clinical utility. Additionally, the application of multi-omics approaches (44) will enable a more comprehensive understanding of the regulatory networks and dynamic changes underlying immune function at the extremes of age.

## 5 Conclusion

This study identifies a critical decoupling between absolute and relative lymphocyte metrics across age extremes: infants exhibit thymus-driven expansion of Tn, while older adults show accumulation of memory T cell subsets and a predominance of NK cells, reflecting divergent immune strategies. The age-polarized CD4^+^/CD8^+^ ratio emerges as a dynamic biomarker of thymic output and immunosenescence. Clinically, prioritizing absolute counts over proportional data may help avoid misinterpretation of immune competence, particularly in pediatric and geriatric monitoring. Although the analysis was limited to peripheral blood, this study provides a framework for precision immunological assessment in individuals at extreme ages, supporting the integration of absolute lymphocyte counts into age-specific vaccination strategies and offering meaningful insights for clinical evaluation, treatment planning, and prognostic assessment.

## Data Availability

The original contributions presented in the study are included in the article/**Supplementary Material**. Further inquiries can be directed to the corresponding authors.
