# Supplementary material for: Decoupled dynamics of absolute and relative lymphocyte counts and age−polarized CD4+/CD8+ ratio in infants versus older adults
Source: Front Immunol. 2025 Jul 24;16:1599515. doi: 10.3389/fimmu.2025.1599515 (PMC12328443; doi:10.3389/fimmu.2025.1599515)
Supplement: Supplementary file 1 [file SupplementaryFile1.docx]

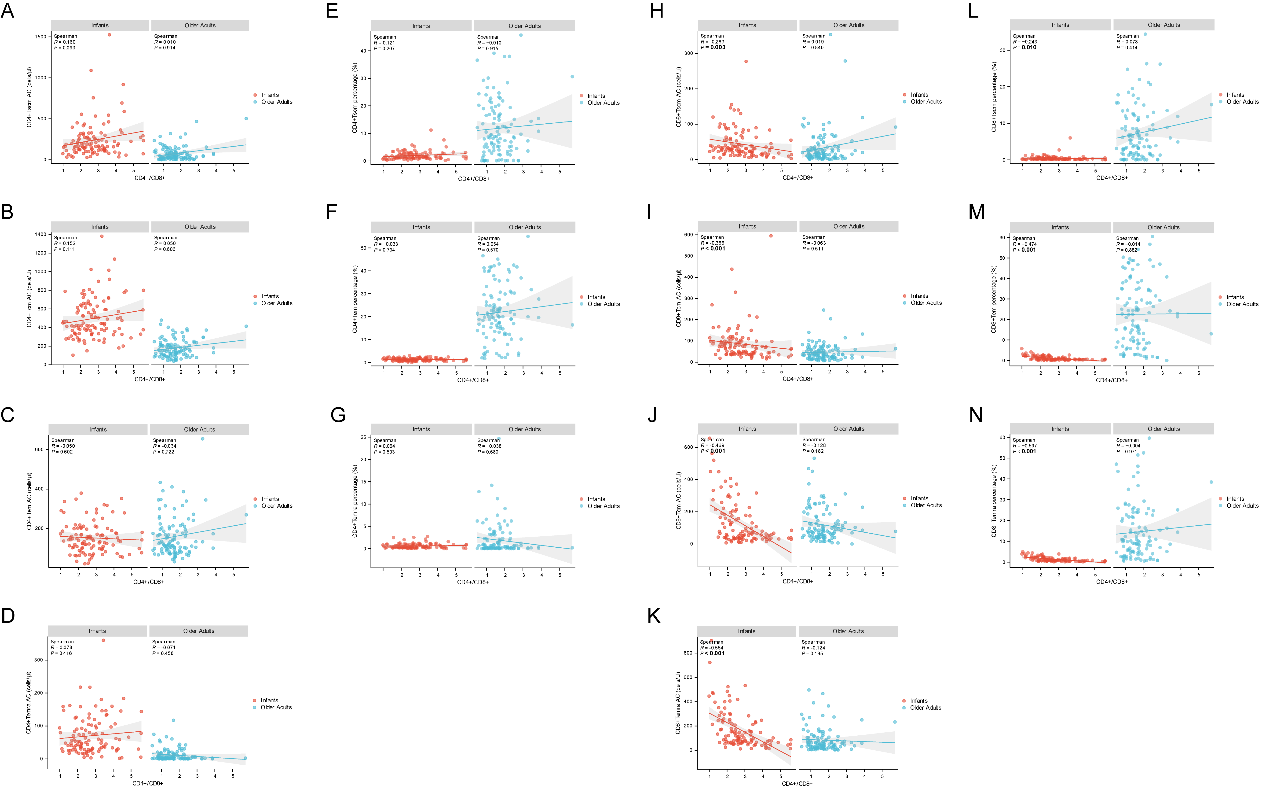


**Supplementary Figure.1** Scatter plots demonstrating the correlations between CD4^+^/CD8^+^ ratio and Tn and Tm lymphocyte subsets at extremes of age. (A-D) Scatter plots demonstrating CD4^+^/CD8^+^ ratio—absolute counts of CD4^+^ Tscm, Tcm, Tem, and Temra correlations. (E-G) Scatter plots demonstrating CD4^+^/CD8^+^ ratio—percentages of CD4^+^ Tscm, Tem, and Temra correlations. (H-K) Scatter plots demonstrating CD4^+^/CD8^+^ ratio—absolute counts of CD8^+^ Tscm, Tcm, Tem, and Temra correlations. (L-N) Scatter plots demonstrating CD4^+^/CD8^+^ ratio—percentages of CD4^+^ Tscm, Tem, and Temra correlations. Correlations were assessed by scatter plot, and the strength of linear correlation was determined by calculating a Pearsonman coefficient. Infants were represented by red dots and older adults by blue dots. The shaded line around each linear fit line represented 95% confidence interval. (**P* < 0.05; ***P* < 0.01; ****P* < 0.001) Absolute counts were denoted by “AC”.
